# Maturation and Phenotypic Heterogeneity of Human CD4+ Regulatory T Cells From Birth to Adulthood and After Allogeneic Stem Cell Transplantation

**DOI:** 10.3389/fimmu.2020.570550

**Published:** 2021-01-18

**Authors:** Tiago R. Matos, Masahiro Hirakawa, Ana C. Alho, Lars Neleman, Luis Graca, Jerome Ritz

**Affiliations:** ^1^ Division of Hematologic Malignancies and Department of Medical Oncology, Dana-Farber Cancer Institute, Boston, MA, United States; ^2^ Harvard Medical School, Boston, MA, United States; ^3^ Instituto de Medicina Molecular, Faculdade de Medicina, Universidade de Lisboa, Lisbon, Portugal; ^4^ Amsterdam University Medical Centers, Department of Dermatology, University of Amsterdam, Amsterdam, Netherlands

**Keywords:** Treg - regulatory T cell, immunology, T cell, heterogeneity, diversity, GvHD, alloHSCT, CD4

## Abstract

CD4^+^ Regulatory T cells (Treg) play a critical role in maintaining immune homeostasis. Various Treg subsets have been identified, however the heterogeneity of Treg subpopulations during development remains uncharacterized. Using mass cytometry we obtained single cell data on expression of 35 functional markers to examine the heterogeneity of Treg cells at birth and in adults. Unsupervised clustering algorithms FlowSOM and ACCENSE were used to quantify Treg heterogeneity. As expected, Treg in umbilical cord blood were predominately naïve while Treg in adult blood were predominately central memory and effector memory cells. Although umbilical cord blood Treg are mostly naïve cells, we observed multiple phenotypic Treg subsets in cord blood. Nevertheless, peripheral blood in adults contained higher percentages of Treg and the heterogeneity of Treg was significantly increased in adults. We also studied Treg heterogeneity throughout a 2-year period after allogeneic hematopoietic stem cell transplantation (alloHSCT) and in patients with chronic graft-versus-host disease (cGVHD). Treg heterogeneity recovered rapidly after alloHSCT and gradually increased in the first two years post-transplant. However, patients with cGVHD had significantly fewer distinct Treg subpopulations, proposing a correlation between a disrupted Treg heterogeneity and cGVHD. Our study is the first to compare human Treg heterogeneity at birth, in healthy adults and in patients after alloHSCT with and without cGVHD. This approach to characterize Treg heterogeneity based on expression of a large panel of functional markers may enable future studies to identify specific Treg defects that contribute to immune dysfunction.

## Highlights

- Distinct subpopulations of CD4^+^ Treg cells are present at birth and phenotypic Treg heterogeneity increases in adulthood
- CD4^+^ Treg heterogeneity increases after allogeneic stem cell transplantation and is reduced during chronic Graft-versus-Host-Disease

## Introduction

Regulatory T cells (Tregs) are essential elements of a healthy immune system. They comprise between 5–10% of the peripheral blood CD4^+^ T cell compartment in healthy individuals and play a critical role protecting their host against immunopathological damage following inflammatory or immunological challenges (1, 2). Tregs are able to suppress a range of effector cell types through several mechanisms, both direct and indirect, ensuring peripheral tolerance and immune homeostasis (3, 4). The intricate balance between immune response and suppression can be disturbed by both a reduced number of Tregs as well as a decline in their functionality, potentially leading to autoimmune pathology (5).

Tregs originate in the thymus and can be identified by expression of CD3, CD4, high levels of surface CD25, low levels of CD127 and intracellular FoxP3 (6). Tregs can be further divided into three main maturation categories: naïve, central memory (CM) and effector memory (EM) (7). Naïve Tregs express high levels of CD45RA and low levels of CD45R0 and FoxP3, whereas memory Tregs are FoxP3^hi^ and CD45RA^-^ (8, 9). In healthy individuals up to 30% of the regulatory T cells are naïve (10). As individuals age, the relative fraction of naïve Tregs decreases due to thymic involution, while the proportion of memory Tregs increases (11). Nevertheless, recent thymic emigrants (RTE), which co-express CD31 and CD45RA, are still present in adults constituting up to 11% of all naive Tregs.

It has been reported that the Treg population in adult peripheral blood contains up to 22 phenotypically distinct subpopulations, thus offering new insights into the heterogeneity of these cells (12). Nevertheless, extensive comparisons between Treg in adult peripheral blood and umbilical cord blood (CB) have not previously been undertaken. Presumably, CB Treg are mostly comprised of a homogeneous population of naïve cells (10, 13, 14). However, even though the majority of CB Tregs are naïve, CB also contains small numbers of memory Tregs, possibly due to prenatal antigen activation (15). It has also been proposed that maternal cells pass the placenta and remain in fetal lymph nodes, where these cells induce the development of fetal Tregs that suppress antimaternal immunity (16). The expansion potential of CB Treg has been shown to be higher than adult Treg, and expanded CB Treg are functionally active (17). This has led to the use of expanded CB Tregs for treatment of GVHD after allo-HSCT (17). However, the extent to which CB Tregs are a homogenous population has not been studied or whether the extensive heterogeneity present in adult Treg is also present in CB Treg.

Mass cytometry by time of flight (CyTOF) allowed us to investigate CB and adult Treg in unprecedented detail by simultaneously detecting and quantifying 35 markers in individual cells (18–20). To quantify heterogeneity and provide more insight into the phenotype of CB Treg, we used FlowSOM and ACCENSE for high dimensional analysis of mass cytometry data. These analytic tools allow us to quantify human Treg heterogeneity based on expression of a large set of activation, proliferation, tissue-homing and functional markers in conjunction with stages of Treg maturation and differentiation.

These tools revealed heterogeneous populations of Treg in both CB and adult blood but CB Treg were less heterogeneous with respect to maturity and functional markers. After allo-HSCT the number of distinct Treg subpopulations gradually increased during a two-year follow-up period. Patients with cGVHD had significantly fewer distinct Treg subpopulations based on functional markers, proposing a correlation between a disrupted Treg heterogeneity and cGVHD.

## Methods

### Donor and Patient Characteristics

Peripheral blood samples were obtained from 14 healthy individuals (8 males and 6 females) with a median age of 44 years (range, 20–69 years), two children (male of 2 years old and female of 10 years old) and from five discarded umbilical cord blood collections (from two males and three females). We also studied peripheral blood from 10 adult patients who underwent allogeneic HSCT at the Dana-Farber Cancer Institute and Brigham and Women’s Hospital, Boston Massachusetts. All transplant patients received reduced intensity conditioning with fludarabine plus busulfan followed by infusion of unmodified G-CSF mobilized peripheral stem cell grafts. No patients received anti-thymocyte globulin for GVHD prophylaxis or low-dose interleukin-2 (IL-2) for treatment of chronic GVHD. Fresh blood samples were obtained at 6 different time points (0, 1, 3, 6, 12, and 24 months) after transplant or during cGVHD (6 months). Patients with relapse were not included. Written informed consent was obtained from patients and healthy donors prior to sample collection, in accordance with the Declaration of Helsinki. Protocol approval was obtained from the Human Subjects Protection Committee of the Dana-Farber/Harvard Cancer Center.

### Sample Preparation

CB mononuclear cells (CBMCs) and PBMCs were isolated from freshly drawn samples by density gradient centrifugation (Ficoll-Paque PLUS; GE Healthcare). Freshly isolated CBMCs and PBMCs from healthy donors were immediately used for antibody staining. PBMCs from patients were washed and cryopreserved in BAMBANKER (Lymphotech) before being analyzed.

### Metal-Tagged Monoclonal Antibodies

A panel of 35 metal-tagged monoclonal antibodies was used for analysis of CBMCs and PBMCs. A list of all antibodies and corresponding metal tags is provided in **Supplementary Table 1**. All pre-conjugated antibodies were purchased from Fluidigm. All other antibodies were purchased in carrier-protein-free PBS and conjugated with the respective metal isotope using the MaxPAR antibody conjugation kit (Fluidigm) according to the manufacturer’s recommended protocol. Metal-labeled antibodies were diluted to 0.5 mg/ml in Candor PBS Antibody Stabilization solution (Candor Bioscience GmbH) for long-term storage at 4°C.

### Antibody Staining for Mass Cytometry

CBMCs and PBMCs were washed with MaxPar Cell Staining Buffer (Fluidigm) and blocked with Human FcR Blocking Reagent (Miltenyi Biotec) for 10 minutes at room temperature. Cells were then incubated with all antibodies targeting cell surface markers for 30 minutes at room temperature and then washed twice with Cell Staining Buffer. After washing, cells were fixed with Cytofix Fixation Buffer (BD Biosciences) and permeabilized with Phosflow Perm Buffer III (BD Biosciences) following the manufacturer’s instructions. Fixed/permeabilized cells were washed twice with Cell Staining Buffer and incubated with all antibodies targeting intracellular antigens for 30 minutes at room temperature. After staining with intracellular antibodies, cells were washed twice with Cell Staining Buffer and incubated with 191/193Ir DNA intercalator (Fluidigm) following the manufacturer’s instructions. Prior to mass cytometry analysis, cells were washed twice with Cell Staining Buffer and twice with MaxPar Water (Fluidigm).

### Mass Cytometry

Cells were analyzed on a CyTOF 2 mass cytometer (Fluidigm) at an event rate of approximately 500 cells/s. To normalize CyTOF data over different days, EQ Four Element Calibration Beads (Fluidigm) were added in all samples. Resulting data were analyzed with software available through Cytobank (www.cytobank.org). To remove debris and doublets, single cells were gated based on cell length and DNA content as described by Bendall et al. (21). To interpret high dimensional single-cell data produced by mass cytometry, we used a visualization tool based on the viSNE algorithm that creates a two-dimensional view of high-dimensional cytometry data at single-cell resolution, making it possible to not only visually identify interesting and rare subsets while preserving nonlinearity, but also to gate single-cell events across different samples (22).

### Gating of Populations

**Figure 1:**
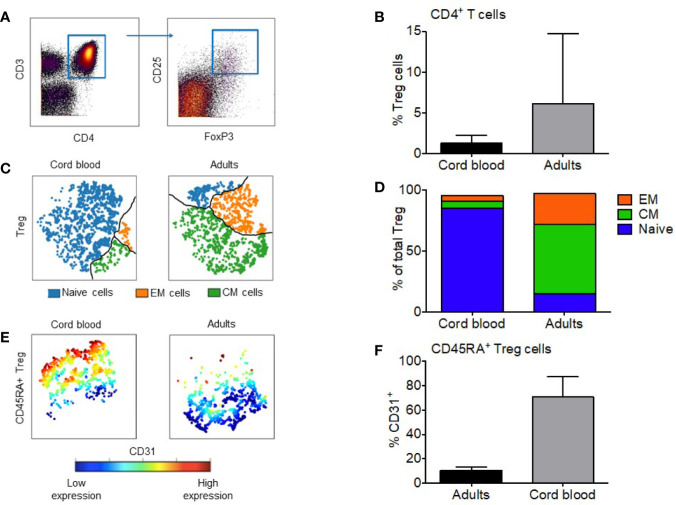
Regulatory T cells from cord blood are mostly CD31+ naive cells. **(A)** Manual gating of Treg based on expression of CD4 and CD25^+^FOXP3^+^ co-expression. **(B)** Median percentage of Tregs in the CD4^+^ compartment of cord blood and adult PBMC; the error bars show the range. **(C)** Representative visual composition of Treg maturation subsets in viSNE maps showing single cell relationships; subsets are divided by the black line: blue = naïve subset; green = Central Memory (CM) subset; orange = Effector Memory (EM) subset. **(D)** Relative composition of Naïve, CM and EM subsets in cord blood and adult Treg. Median values are shown for five CB and 14 adult Treg samples. **(E)** Expression of CD31 visualized with viSNE maps within naïve fraction of CB and adult Treg. Cells are colored according to intensity of CD31 expression. **(F)** Median percentage of naïve Treg cells expressing CD31^+^, the error bars show the range. * represents statistical significance (p-value < 0.05). In ViSNE maps, each point represents a single cell.

Treg were defined by CD25^+^FOXP3^+^ co-expression (**Figure 1A**). Naive cells were gated from the Treg population with the expression of CD45RA^+^CD62L^+^, CM as CD45RA^-^CD62L^+^, and EM as CD45RA^-^CD62L^-^. Recent thymic emigration (RTE) cells were gated by co-expression of CD45RA and CD31 (23). Each sample was gated manually *via* Cytobank.

### Clustering Analysis

FlowSOM (R package accessible within the www.bioconductor.org platform) was used for automated clustering. FlowSOM was run in R Studio and allows for unsupervised clustering and dimensionality reduction of data obtained from mass cytometry. Subsequent subpopulation analysis was repeated in ACCENSE (standalone application accessible from http://www.cellaccense.com/) for reliability purposes. ACCENSE is a tool for exploratory analysis of high-dimensional single-cell data such as that generated by mass cytometry (24). By combining a nonlinear dimensionality reduction algorithm (t-SNE) with a k-means clustering algorithm both visualization for exploratory analysis and automated cell classification into subpopulations is performed. Subpopulation quantification was completed twice based on 26 functional markers and 6 maturity markers from the cytometry panel. ACCENSE was also used to make subsequent visual figure maps. **Supplementary Table 2** shows which markers were used.

### Statistical Analysis

Graphpad Prism 7.04 was used for data analysis. Mann-Whitney test was used to compare unpaired populations. The Wilcoxon signed-rank test was used to compare paired samples for continuous variables and expression levels of proteins between subpopulations and between different time points. All tests were 2-sided at the significance level of 0.05 and multiple comparisons were not considered.

## Results

### Prevalence of Treg Maturation Subsets at Birth and Adulthood

To compare the maturity of the Treg cell compartment between cord blood and adult blood we gated the single cell data biaxially in Cytobank to distinguish Tregs in all samples (**Figure 1A**). Treg were classified by the co-expression of intracellular FoxP3 and high-expression of surface CD25 (6). For complete visualization of the gating strategy see **Supplementary Figure 1**. The median percentage of regulatory T cells in adult blood was a 4.6-fold higher than cord blood (6.0% vs. 1.3%; **Figure 1B**) (p < 0.0001). Specific maturation subsets were defined as follows: naïve (CD45RA^+^CD62L^+^), CM (CD45RA^-^CD62L^+^), and EM (CD45RA^-^CD62L^-^) (7–9). **Figure 1C** identifies these three distinct populations within the Treg compartment and visually represents the difference between CBMC and adult PBMC. Naïve Treg cells make up the largest subset in CB at 85.2%, followed by CM cells at 5.6% and EM at 4.5%. In adult PBMC, CM Treg form the major subset at 56.5%, followed by EM (25.2%) and naïve cells (15.2%) (**Figure 1D**). We also quantified the fraction of recent thymic emigrants (RTE) within the naïve Treg subset. RTE can be gated from the naïve population *via* the expression of CD31, seen in **Figure 1E**. Within CB Treg, a median of 70.7% of gated CD45RA^+^ cells expressed CD31. In adult PB Treg, a median of 10.19% of gated CD45RA^+^ cells expressed CD31 (p = 0.0002) (**Figure 1F**).

### CD4 Treg Cell Heterogeneity Is Established in Umbilical Cord Blood and Increases With Age

**Figure 2:**
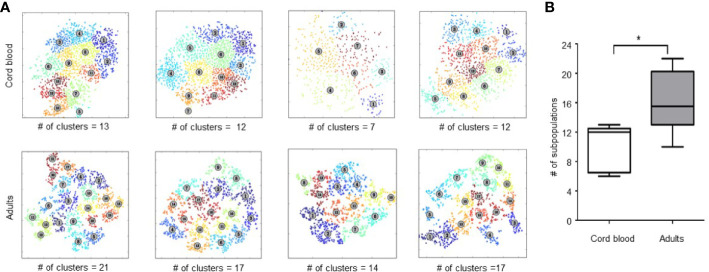
Analysis of Treg heterogeneity by unsupervised clustering based on expression of 26 functional markers. **(A)** Treg metaclusters identified by ACCENSE in four representative samples of cord blood (CB) and four adult Treg samples. Each point represents one cell, the color of the cells denotes a specific cluster. The number of metaclusters identified in each sample is shown below each box. **(B)** Phenotypic heterogeneity of Treg in CB and adult PB. Five CB and 14 adult PB samples were studied. The center bar in the box is the median; Whiskers illustrate the minimum and maximum values obtained. * represents statistical significance (p < 0.05).

We then examined the phenotypic heterogeneity of Treg in CB and adult peripheral blood by clustering Tregs based on the expression of 26 functional markers. To quantify heterogeneity within Treg, we used unsupervised cluster analysis with FlowSOM. Weber et al, 2016. previously compared 13 flow and mass cytometry clustering tools, recommending that FlowSOM (with optimal meta-clustering but without automatic selection of number of clusters) be used as a first choice for analyzing new data sets (25). We replicated this analysis with our 26-marker panel and results were further validated by a separate analysis tool, ACCENSE. **Figure 2A** shows Treg cell clustering on functional markers included in our panel for 4 CB and 4 adult PB samples. Unsupervised clustering based on all 26 marker parameters revealed a median of 15.5 distinct clusters in adult Treg (range, 10–22) and 12 Treg clusters in CB Treg (range, 6–13) (p = 0.008) (**Figure 2B**). Although heterogeneity of CB Treg is substantial this heterogeneity increases significantly in adults. There was no variation of number of clusters within healthy controls regarding their age. In fact, we analyzed Treg heterogeneity in two children (2 and 10 years old), and both had 16 and 18 clusters, respectively (**Supplementary Figure 2**). This finding suggests that Treg heterogeneity is acquired very early in life.

### CD4 Treg Cell Maturation Heterogeneity Also Increases Between Birth and Adulthood

**Figure 3:**
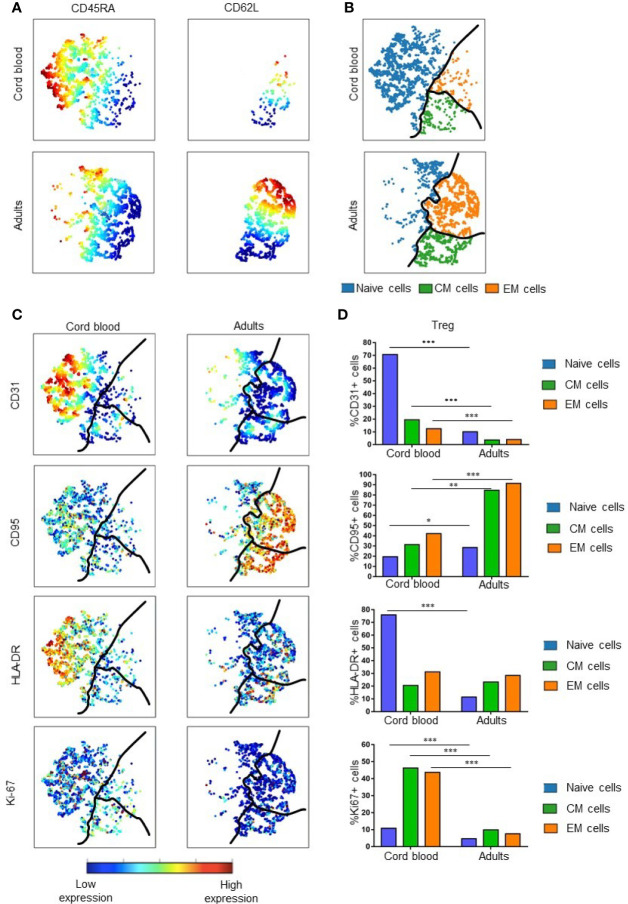
Treg maturation in cord blood and adult peripheral blood. **(A)** Expression of CD45RA and CD62L in cord blood and adult Treg. Color indicates the level of expression of the labeled marker in viSNE maps from two representative samples. **(B)** Visual representation of Treg maturation in viSNE maps based on expression of CD45RA and CD62L. Subsets are divided by the black line: blue = naïve Treg; green = Central Memory (CM) Treg; orange = Effector Memory (EM) Treg. **(C)** Expression of specific functional markers in representative examples of cord blood and adult Treg. **(D)** Median percentage of marker positive cells within naïve, CM and EM Treg subsets. Results are compared for five cord blood Treg samples and 14 adult Treg samples. * represents statistical significance (*p < 0.05, **p < 0.005, ***p < 0.0005). In viSNE maps, each point represents a single cell. Cells are colored according to intensity of expression of the indicated marker (excluding B).

After establishing that heterogeneity of Treg in umbilical cord blood increases in adulthood, we further analyzed phenotypic heterogeneity related to levels of Treg maturity within naïve, CM and EM Treg subpopulations. **Figures 3A, B** show the gating strategy using CD45RA and CD62L to identify the naïve/CM/EM subsets in a viSNE map. This allowed us to visualize and quantify the following phenotypic and functional markers in each subset: CD31 (recent thymic emigrant marker), Ki-67 (marker for proliferation), CD95 (marker of extrinsic pathway apoptosis), and HLA-DR (activation marker) (**Figures 3C, D**).

The percentage of CD31^+^ Tregs within the naïve subset of CB was significantly higher than in naïve adult Tregs (p = 0.0002). These results are consistent with the previously established higher expression of CD31 in CB cells compared to adult Tregs. In contrast, all 3 maturation subsets of adult Tregs express significantly more CD95 than corresponding CB Treg subpopulations. Although few naïve Tregs express CD95, the expression of this marker was also significantly different (p = 0.01) between CB an adult Treg. Larger differences were seen when comparing CD95 expression in CM and EM Treg populations in CB and adults (p = 0.005 and 0.0002, respectively). This suggests that more memory Tregs are susceptible to apoptosis in adult than in CB Tregs. With regard to the activation marker HLA-DR, naïve CB Tregs expressed higher levels of HLA-DR than naïve adult Tregs (p = 0.0002). This suggests that naïve Tregs are the most activated subset within CB. There was little difference in expression of HLA-DR in CM and EM Tregs of newborns and adults, perhaps indicating that these subsets have similar activity in both age groups. Ki-67 is expressed at higher levels in all CB Treg subsets, indicating that all CB Treg cells proliferate at a higher rate than their adult counterparts. For each of the subgroups, significant differences were found, with p = 0.0003 for naïve, p = 0.0002 for CM and p = 0.0002 for EM Tregs.

### Differential Expression of Markers in Distinct Populations Reveal Functional Characteristics

**Figure 4:**
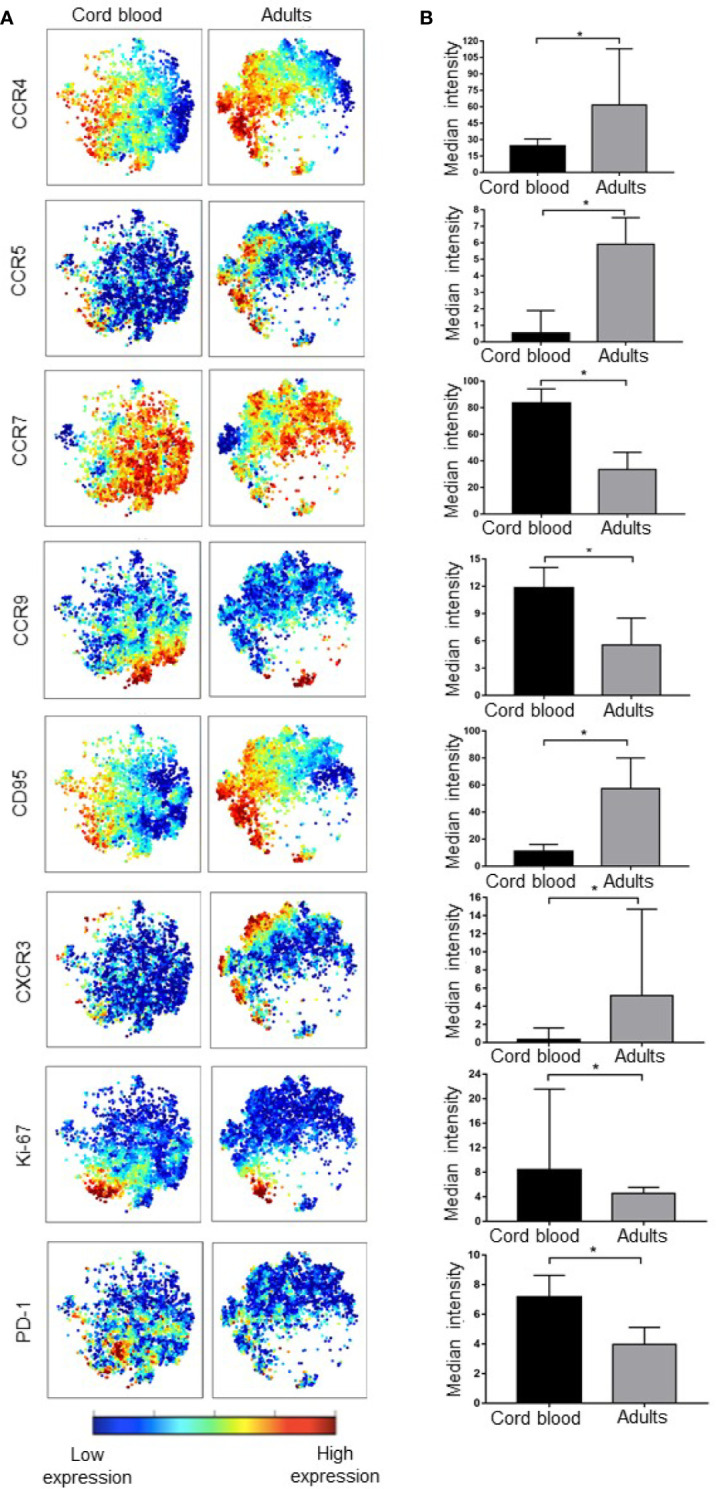
Cord blood and adult peripheral T regulatory cells differ in expression of various markers. **(A)** viSNE maps show the expression of functional Treg markers in cord blood and adult PBMC, color indicates the level of expression of the labeled marker. **(B)** Bar graphs matching the viSNE representation to their left showing median expression intensity for each marker, the error bars show the IQ range. * represents statistical significance (p < 0.05). In ViSNE maps, each point represents a single cell. Cells are colored according to intensity of expression of the indicated marker.

Even though our method of unsupervised clustering did not allow us to directly compare expression of markers in different metaclusters, we analyzed the expression of all functional markers in CB versus adult PBMC. From the 26 functional markers (**Supplementary Figure 3**), eight showed significantly different levels of expression between umbilical cord blood and adult peripheral blood. CB Treg cells expressed higher levels of Ki-67 (p = 0.0093) (a nuclear proliferation marker), PD-1 (p = 0.0485) (a marker for reduced apoptosis and exhaustion), CCR9 (p = 0.0196) (a chemokine receptor that regulates lymphocyte trafficking to the small intestine), and CCR7 (p = 0.0036) (a chemokine receptor that regulates lymphocyte trafficking to lymph nodes). On the other hand, adults showed higher expression levels of CCR4 (p = 0.0196) (a chemokine receptor that regulates lymphocyte trafficking to skin), CCR5 (p = 0.0021) and CXCR3 (p = 0.0010) (chemokine receptors that regulates lymphocyte trafficking to inflamed tissues), and CD95 (p = 0.0273) (a marker of extrinsic pathway apoptosis) (**Figure 4**).

Other functional marker differences between CB and adult PBMC that were of interest included CTLA-4 (p = 0.0624), CLA (p = 0.0800), Tbet (p = 0.0800), ICOS (p = 0.0800), and PDL-1 (p = 0.0800), which were slightly lower in adult Tregs.

### Treg Cell Heterogeneity Increases After Allogeneic Hematopoietic Stem Cell Transplantation

**Figure 5:**
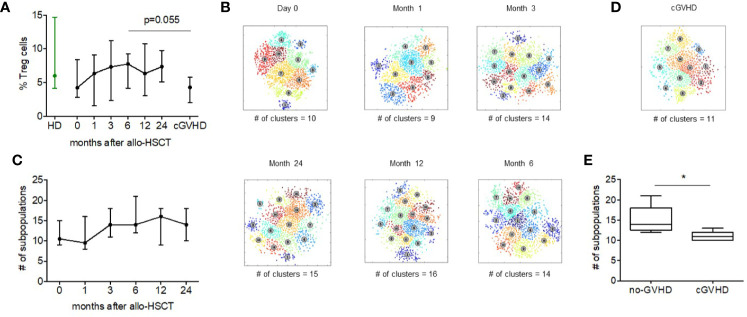
Regulatory T cell heterogeneity after alloHSCT and during cGVHD. **(A)** The median Treg percentage within the CD4^+^ compartment in healthy donors, before and after alloHSCT and in patients with chronic GVHD 6 months after alloHSCT. Error bars show the range of values. **(B)** Treg metaclusters after alloHSCT identified by ACCENSE, based on expression of 26 functional markers. Each point represents one cell and colors denote individual clusters. Results shown are for a representative patient and the number of metaclusers at each time point is shown below each figure. **(C)** The number of Treg metaclusters identified before and after alloHSCT. Results shown are median values for five patients without acute or chronic GVHD after transplant. Error bars show range for values for each time point. **(D)** Treg metaclusters in a representative patient with chronic GVHD 6 months after alloHSCT. Colors denote individual clusters identified by ACCENSE. **(E)** Treg heterogeneity in patients with and without chronic GVHD 6 months after alloHSCT. Box and whisker plots show the number of Treg metaclusters based on expression of 26 functional markers. The center bar in the box is the median. Whiskers illustrate the minimum and maximum values.

Acknowledging the important role of regulatory T cells in allogeneic hematopoietic stem cell transplantation (alloHSCT) and following our previous studies describing the reconstitution of Treg after alloHSCT (23), we examined the heterogeneity of Treg subsets at various times after alloHSCT. Using the same mass cytometry panel, we analyzed Tregs from five patients that received unmodified peripheral blood stem cell grafts at 6 time points: Day 0 and days 1, 3, 6, 12, and 24 months after transplant. None of these patients developed acute or chronic GVHD during this time period. The percentage of CD4^+^ Treg cells increased gradually in the first 6 months after alloHSCT and subsequently remained relative stable throughout the 2-year follow up period (**Figure 5A**).

We subsequently examined Treg heterogeneity during this period (**Figures 5B, C**). Compared to Treg pre transplant, there was a decrease in the number of Treg subsets 1 month after transplant. We then observed an increase from a median of 9.5 subpopulations to 14 subpopulations 3 months after transplant. This level of Treg heterogeneity remained relatively stable from 3 to 24 months after transplant in this group of patients who did not develop acute or chronic GVHD. Interestingly, only a few markers vary their expression noticeably since the transplant. D49a, GITR, CTLA-4, CXCR3, Tim-3, CCR7, CD28, and CCR4 are 2-fold more expressed at day 0 compared to after month 1. From month 3 onwards the expression of the markers remains constant among alloHSCT patients and similar to adult Treg cells.

### Treg Cell Heterogeneity is Reduced During cGVHD

Previous studies have shown that patients with active chronic GVHD have a lower frequency of Treg cells (22, 26). To determine whether this quantitative Treg deficiency was also associated with abnormal Treg heterogeneity we examined peripheral blood from five patients with chronic GVHD and compared results to samples obtained 6 months after alloHSCT from five patients without GVHD that had been previously analyzed. Gating on CD4+FoxP3+ Treg, we first compared Treg percentages in patients with and without cGVHD. Median %Treg was 4.28 (range, 2.03–6.77) in patients with cGVHD compared to 7.74 (range, 4.18–9.27) in patients without cGVHD (p = 0.055). Using FlowSOM, we compared Treg heterogeneity in samples from patients with and without cGVHD. Treg from patients with cGVHD were found to have 11 Treg metaclusters (range, 10–13) compared to 14 metaclusters (range, 12–21) in patients without cGVHD (p = 0.02) (**Figures 5D, E**). Although our method of unsupervised clustering did not identify specific characteristics of missing metaclusters in cGVHD patients, comparison of marker expression found a significant difference in expression of Helios and CLA between people with and without cGVHD (**Supplementary Figure 4**). Helios was expressed at higher levels in patients without cGVHD while the expression of CLA was higher in cGVHD patients. PD-1, CCR4 and HLA-DR also were highly expressed (non-significant) in non-cGVHD patients. For CD49a, CCR5, CD62L, BCL-2, and CD39 the opposite was observed, with a non-significant but higher expression in cGVHD patients.

## Discussion

Using mass cytometry and advanced computational algorithms to simultaneously measure expression of 35 phenotypic and functional markers in individual cells, we undertook a detailed analysis of Treg heterogeneity in humans. Our study initially focused on Treg in umbilical cord blood and peripheral blood in healthy adults and subsequently was expanded to include Tregs reconstituting after allogeneic HSCT and in patients with cGVHD. Unsupervised clustering algorithms based on 26 functional markers were used to establish the number of Treg metaclusters in individual samples and thereby quantify heterogeneity within the Treg population in each sample at each time point. This resulted in a unique examination of Treg heterogeneity throughout life and through a critical period where Treg are known to play an important role in immune reconstitution and the establishment of immune tolerance.

At birth, CB Tregs are predominately comprised of naïve cells (13, 14). Genomic diversity and proliferative capacity of CB Treg is very high (17), and this has facilitated the use of CB-derived *in vitro* expanded Treg for treatment or prevention of GVHD after alloHSCT (27). Our studies confirmed that CB Treg are predominately naïve cells, with a high fraction of recent thymic emigrants. In contrast, adult Treg are more mature, being predominately CM and EM cells with a much smaller fractions of naïve cells and recent thymic emigrants. Nevertheless, CB Treg were found to be relatively heterogeneous reflecting variable states of differentiation, maturation and activation, despite being predominately naïve cells. Treg heterogeneity increased significantly in healthy adults reflecting past exposures and additional levels of differentiation, maturation and activation *in vivo*.

To assess heterogeneity within defined stages of Treg maturation we compared the expression of CD31 (recent thymus emigrant), Ki-67 (proliferation), CD95 (apoptosis), and HLA-DR (functional activation) in naïve, CM, and EM Treg. The frequency of CD31^+^ Tregs in the naïve CB Treg was significantly higher than in naïve adult Treg. This likely reflects decreased thymic function and increased homeostatic expansion of naïve T cells in adults. We also observed increased expression of CD95 in naïve, CM and EM subpopulations in adult Tregs compared to CB Treg. This likely reflects higher levels of exhaustion and terminal differentiation in adult Treg. In contrast, HLA-DR was more highly expressed naïve CB Treg and Ki-67 was more highly expressed at all levels of differentiation in CB Treg, indicating that all subsets of CB Treg are activated and highly proliferative.

In patients who undergo alloHSCT, recipient T cells are rapidly replaced as donor cells engraft and reconstitute a fully functional immune system in the recipient. Proper functioning of the donor immune system requires balanced recovery of regulatory elements as well as effector cells and the development of cGVHD can be predicted by impaired recovery of Treg leading to an abnormally low ratio of Treg to conventional effector T cells (28). Enhancement of Treg recovery through Treg infusions or administration of low dose IL-2 to selectively induce expansion of Treg *in vivo* can prevent or treat cGVHD progression (27, 29–33). The ability to manipulate Treg after alloHSCT and in patients with autoimmune diseases has sparked interest in the development of Treg-directed therapies. However, there has been relatively little consideration of the potential importance of functional Treg heterogeneity in addition to the ability to simply increase Treg counts *in vivo*. In our analysis of Treg heterogeneity in adult patients after transplant we found that Treg heterogeneity recovered rapidly in patients without acute or chronic GVHD. By 3–6 months after alloHSCT, levels of Treg heterogeneity were similar to healthy adults. However, the number of Treg metaclusters in patients with cGVHD at 6 months was significantly decreased compared to patients without cGVHD. These findings suggest that lack of functional Treg subsets may contribute to the development of cGVHD in addition to a simple numerical deficiency in this setting. High T cell receptor (TCR) diversity has been correlated with establishment and maintenance of self-tolerance (34) and required for optimal suppressive function of Treg cells in murine models of GVHD (35). Hence, future studies should correlate the TCR repertoire diversity to Treg subpopulations in order to determine whether subpopulations share a clonal origin.

In summary, our findings reveal considerable heterogeneity of Treg subsets that is not detected in routine characterization of Treg by flow cytometry with a limited set of markers. We also show that Treg heterogeneity varies considerably among individuals and in patients after alloHSCT. Although this heterogeneity is based on the variable expression of functional markers, further studies are needed to establish the extent to which this phenotypic heterogeneity reflects actual functional differences between different Treg metaclusters and the ability of different Treg metaclusters to regulate different immune cells and immune networks *in vivo*. The main limitation of our study was the inability to define each individual cluster and compare them between samples. Since Treg are known to be capable of functional plasticity it will also be important to examine the stability of distinct metaclusters *in vitro* and *in vivo*. As Treg directed therapies are evaluated in patients with autoimmune diseases as well as GVHD, it may be important to examine the effects of these interventions on Treg heterogeneity as well as the number of circulating Treg.

## Data Availability Statement

The original contributions presented in the study are included in the article/**Supplementary Material**; further inquiries can be directed to the corresponding author.

## Ethics Statement

Written informed consent was obtained from patients and healthy donors prior to sample collection, in accordance with the Declaration of Helsinki. Protocol approval was obtained from the Human Subjects Protection Committee of the Dana-Farber/Harvard Cancer Center. Written informed consent to participate in this study was provided by the participants’ legal guardian/next of kin.

## Author Contributions

TM designed the research studies, conducted the experiments, acquired and analyzed the data, and wrote the manuscript. MH and AA conducted the experiments, acquired and analyzed the data, and edited the manuscript. LN analyzed the data and edited the manuscript. LG and JR designed the research studies, analyzed the data, and edited the manuscript. All authors contributed to the article and approved the submitted version.

## Funding

This work was supported by NIH grant P01CA229092, EADV Research Fellowship, Rene-Touraine Fellowship and a generous contribution from the Fundação para a Ciência e a Tecnologia (FCT), FCT SFRH/BD/98980/2013.

## Conflict of Interest

JR receives research funding from Amgen, Equillium, and Kite/Gilead and serves on Data Safety Monitoring Committees for AvroBio and Scientific Advisory Boards for Falcon Therapeutics, LifeVault Bio, Rheos Medicines, Talaris Therapeutics, and TScan Therapeutics. The other authors have no competing financial interest.

The remaining authors declare that the research was conducted in the absence of any commercial or financial relationships that could be construed as a potential conflict of interest.

## Supplementary Material

Supplementary Figure 1 CyTOF gating strategy. **(A)** The gating strategy shown was used on all data prior to downstream analysis, manual gating was performed in Cytobank.

Supplementary Figure 2 Children have distinct regulatory T cells subsets based on all 26 functional markers. Treg subpopulation clusters made with ACCENSE showing 2 representative samples of children (2 years old and 10 years old) peripheral blood samples. Each point represents one cell, the color of the cells denotes a specific cluster.

Supplementary Figure 3 Functional marker expression comparison of cord blood and adult PBMC. **(A)** Heatmaps with marker expression from low (black) to high (yellow). **(B)** Bar graphs showing the median intensity range. * represents statistical significance (p-value < 0.05).

Supplementary Figure 4 Functional marker discrepancies between AlloHSCT and cGVHD. Samples arranged by median intensity and comparing alloHSCT 6 months after transplants and cGVHD samples 6 months post-transplant, the error bars show the IQ range. * represents statistical significance (p-value < 0.05).
