# Supplementary material for: Maturation and Phenotypic Heterogeneity of Human CD4+ Regulatory T Cells From Birth to Adulthood and After Allogeneic Stem Cell Transplantation
Source: Front Immunol. 2021 Jan 18;11:570550. doi: 10.3389/fimmu.2020.570550 (PMC7848157; doi:10.3389/fimmu.2020.570550)
Supplement: Supplementary Figure 1 — CyTOF gating strategy. (A) The gating strategy shown was used on all data prior to downstream analysis, manual gating was performed in Cytobank. [file DataSheet_1.docx]

**SUPPLEMENTAL MATERIALS**

**Supplemental Table 1.** Mass cytometry antibody-conjugate panel. *Antibody was kindly provided by Prof. Eugene Butcher, Butcher Laboratory, Stanford University.

|  | **TARGET** | **CLONE** | **ISOTOPE** | **MANUFACTURER** |
| --- | --- | --- | --- | --- |
| 1 | CD3 | UCHT1 | 170Er | Fluidigm |
| 2 | CD4 | SK3 | 174Yb | Fluidigm |
| 3 | CD25 | 2A3 | 149Sm | Fluidigm |
| 4 | CD127 | A019D5 | 176Yb | Fluidigm |
| 5 | CD8 | RPA T8 | 146Nd | Biolegend |
| 6 | CD45RA | HI100 | 169Tm | Fluidigm |
| 7 | CD31 | WM59 | 145Nd | Fluidigm |
| 8 | CD28 | CD28.2 | 148Nd | Biolegend |
| 9 | HLA-DR | L243 | 141Pr | BioLegend |
| 10 | CD62L | DREG-56 | 153Eu | Fluidigm |
| 11 | CD197 (CCR7) | G043H7 | 159Tb | Fluidigm |
| 12 | CD194 (CCR4) | 205410 | 158Gd | Fluidigm |
| 13 | ACT-1 (α4β7) | - | 163Dy | * |
| 14 | PD-1 | EH12.2H7 | 167Er | Biolegend |
| 15 | CD95 | DX2 | 164Dy | Fluidigm |
| 16 | CD195 (CCR5) | NP-6G4 | 144Nd | Fluidigm |
| 17 | CD39 | A1 | 160Gd | Fluidigm |
| 18 | CD278 (ICOS) | C398.4A | 154Sm | Biolegend |
| 19 | CD183 (CXCR3) | G025H7 | 142Nd | Biolegend |
| 20 | CD49a (VLA1) | [TS2/7](http://www.biolegend.com/index.php?page=pro_sub_cat&action=search_clone&criteria=TS2%2F7) | 162Dy | BioLegend |
| 21 | Tim-3 | F38-2E2 | 150Nd | Biolegend |
| 22 | CD152 (CTLA-4) | 14D3 | 152Sm | eBioscience |
| 23 | CD274 (PDL-1) | 29E.2A3 | 172Yb | Biolegend |
| 24 | GITR | 621 | 166Er | Biolegend |
| 25 | CD199 (CCR9) | [L053E8](http://www.biolegend.com/index.php?page=pro_sub_cat&action=search_clone&criteria=L053E8) | 168Er | BioLegend |
| 26 | CLA | HECA-452 | 143Nd | Biolegend |
| 27 | FOXP3 | PCH101 | 165Ho | eBioscience |
| 28 | Helios | 22F6 | 156Gd | BioLegend |
| 29 | Tbet | 4B10 | 175Lu | Biolegend |
| 30 | Granzyme B | GB11 | 147Sm | [Thermo Fisher Scientific](https://www.thermofisher.com/antibody/product/Granzyme-B-Antibody-clone-GB11-Monoclonal/MA1-80734) |
| 31 | BCL-2 | Bcl-2/100 | 171Yb | BD Biosciences |
| 32 | Ki-67 | B56 | 151Eu | BD Biosciences |
| 33 | DNA | UCHT1 | 103Rh | Fluidigm |
| 34 | DNA | SK3 | 191Ir | Fluidigm |
| 35 | DNA | 2A3 | 193Ir | Fluidigm |

**Supplemental Table 2. FlowSOM and ACCENSE markers.** List of markers used for unsupervised clustering.

|  | **TARGET** | **CLONE** | **ISOTOPE** | **MANUFACTURER** |
| --- | --- | --- | --- | --- |
| 1 | CD45RA | HI100 | 169Tm | Fluidigm |
| 2 | CD31 | WM59 | 145Nd | Fluidigm |
| 3 | CD28 | CD28.2 | 148Nd | Biolegend |
| 4 | HLA-DR | L243 | 141Pr | BioLegend |
| 5 | CD62L | DREG-56 | 153Eu | Fluidigm |
| 6 | CD197 (CCR7) | G043H7 | 159Tb | Fluidigm |
| 7 | CD194 (CCR4) | 205410 | 158Gd | Fluidigm |
| 8 | ACT-1 (α4β7) | - | 163Dy | * |
| 9 | PD-1 | EH12.2H7 | 167Er | Biolegend |
| 10 | CD95 | DX2 | 164Dy | Fluidigm |
| 11 | CD195 (CCR5) | NP-6G4 | 144Nd | Fluidigm |
| 12 | CD39 | A1 | 160Gd | Fluidigm |
| 13 | CD278 (ICOS) | C398.4A | 154Sm | Biolegend |
| 14 | CD183 (CXCR3) | G025H7 | 142Nd | Biolegend |
| 15 | CD49a (VLA1) | [TS2/7](http://www.biolegend.com/index.php?page=pro_sub_cat&action=search_clone&criteria=TS2%2F7) | 162Dy | BioLegend |
| 16 | Tim-3 | F38-2E2 | 150Nd | Biolegend |
| 17 | CD152 (CTLA-4) | 14D3 | 152Sm | eBioscience |
| 18 | CD274 (PDL-1) | 29E.2A3 | 172Yb | Biolegend |
| 19 | GITR | 621 | 166Er | Biolegend |
| 20 | CD199 (CCR9) | [L053E8](http://www.biolegend.com/index.php?page=pro_sub_cat&action=search_clone&criteria=L053E8) | 168Er | BioLegend |
| 21 | CLA | HECA-452 | 143Nd | Biolegend |
| 22 | Helios | 22F6 | 156Gd | BioLegend |
| 23 | Tbet | 4B10 | 175Lu | Biolegend |
| 24 | BCL-2 | Bcl-2/100 | 171Yb | BD Biosciences |
| 25 | Ki-67 | B56 | 151Eu | BD Biosciences |
| 26 | Granzyme B | GB11 | 147Sm | [Thermo Fisher Scientific](https://www.thermofisher.com/antibody/product/Granzyme-B-Antibody-clone-GB11-Monoclonal/MA1-80734) |

**Supplemental Figure 1. CyTOF gating strategy. (A)** The gating strategy shown was used on all data prior to downstream analysis, manual gating was performed in Cytobank.


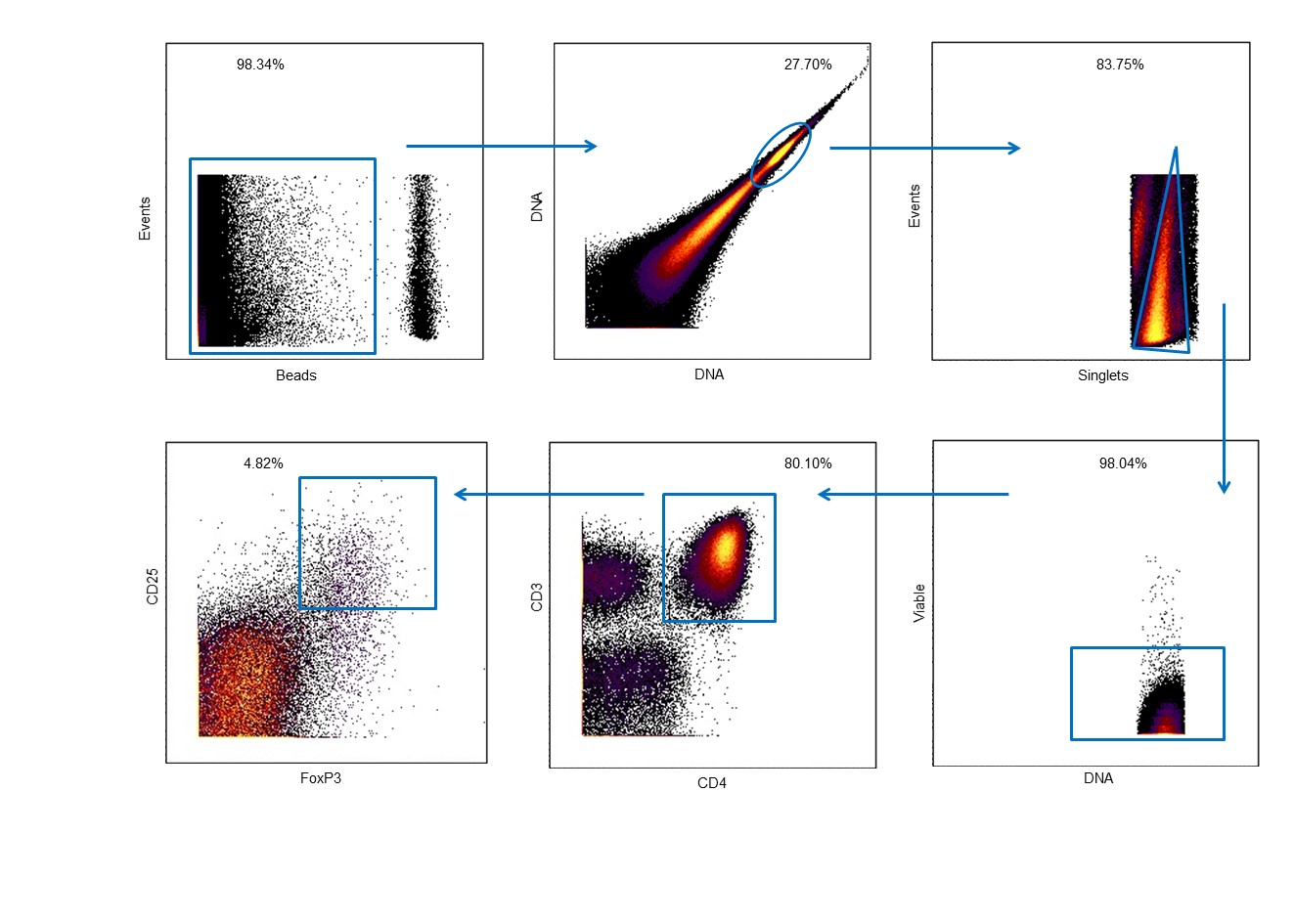


**Supplemental Figure 2. Children have distinct regulatory T cells subsets based on all 26 functional markers.** Treg subpopulation clusters made with ACCENSE showing 2 representative samples of children (2 years old and 10 years old) peripheral blood samples. Each point represents one cell, the color of the cells denotes a specific cluster.
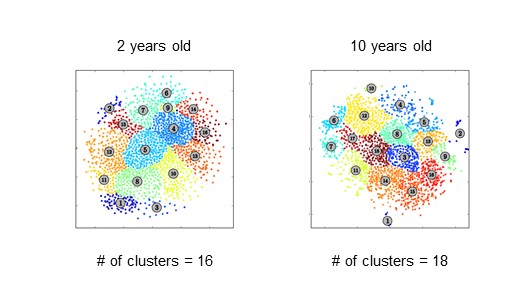


**Supplemental Figure 3. Functional marker expression comparison of cord blood and adult PBMC.** **(A)** Heatmaps with marker expression from low (black) to high (yellow). **(B)** Bar graphs showing the median intensity range. * represents statistical significance (p-value < 0.05).


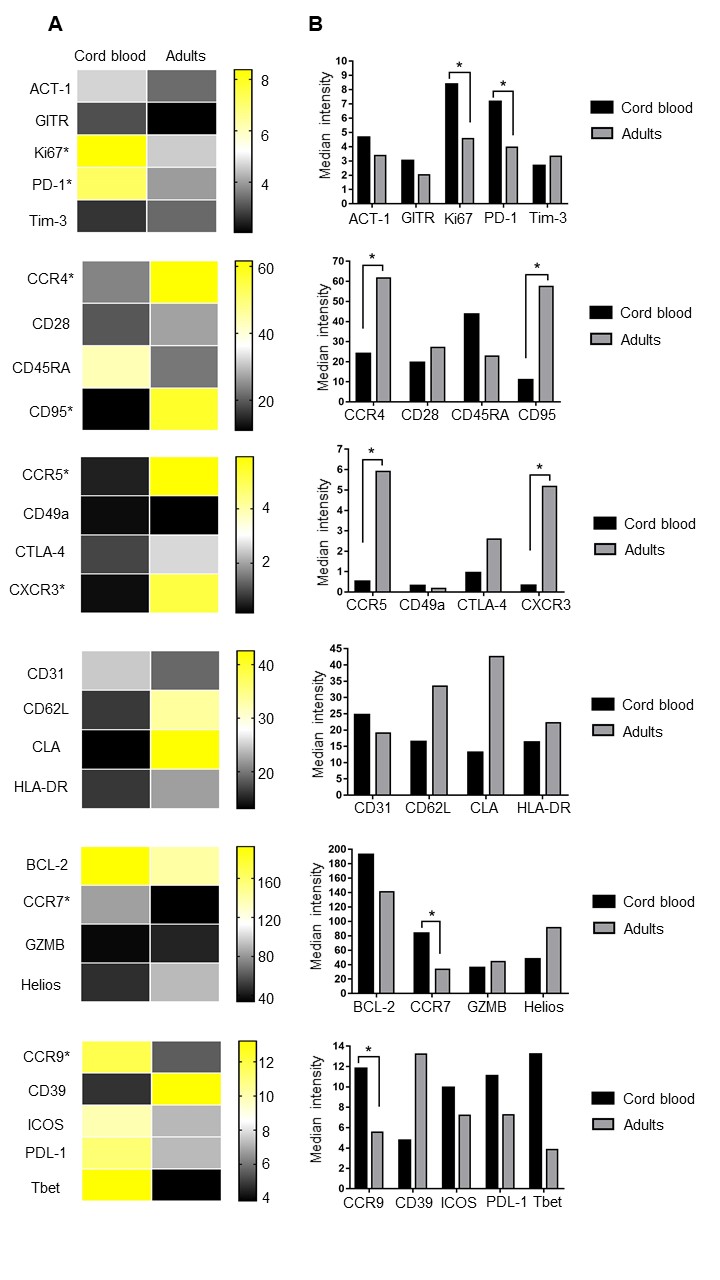


**Supplemental Figure 4. Functional marker discrepancies between AlloHSCT and cGVHD.** Samples arranged by median intensity and comparing alloHSCT 6 months after transplants and cGVHD samples 6 months post-transplant, the error bars show the IQ range. * represents statistical significance (p-value < 0.05).
